# Randomized Controlled Trials on Intracerebral Hemorrhage: A Cross Sectional Retrospective Analysis of CONSORT Item Adherence

**DOI:** 10.3389/fneur.2019.00991

**Published:** 2019-09-20

**Authors:** Kirstin Jauch, Ana Kowark, Mark Coburn, Hans Clusmann, Anke Höllig

**Affiliations:** ^1^Department of Neurosurgery, University Hospital Aachen, RWTH Aachen University, Aachen, Germany; ^2^Department of Anaesthesiology, University Hospital Aachen, RWTH Aachen University, Aachen, Germany

**Keywords:** hemorrhagic stroke, intracerebral hemorrhage, CONSORT statement, randomized controlled trials, transparent reporting

## Abstract

**Object:** Intracranial hemorrhage (ICH) is the second most common cause of stroke but still there is little consolidated knowledge about the optimal treatment strategies (e.g., the benefit of surgical evacuation). We evaluated the current randomized controlled trials (RCTs) on primary ICH (01.2013–03.2017) according to their fulfillment of the CONSORT statement's criteria (published in 2010) –as a marker of transparency and quality of study planning and realization.

**Methods:** A Pubmed and a Cochrane database (including clinicaltrials.gov) search was carried out (01.2014–3.2017, respectively 01.2013–12.2013). Abstracts were screened for inclusion. Eligible full text manuscripts were assessed for the implementation of the CONSORT criteria. Citation frequencies and impact factors of the journals were related to ratio of CONSORT criteria fulfillment. Further, the risk of bias according to the Risk of bias tool 2 (RoB 2) was assessed.

**Results:** Overall 3097 abstracts were screened for inclusion; 39 studies were suitable for final analysis. A mean fulfillment ratio of 51% (±28%) was found. A high correlation between impact factor and adherence to CONSORT criteria was shown (*r* = 0.7664; *p* < 0.0001). Citation frequency per year was related to ratio of CONSORT item fulfillment (*r* = 0.6747; *p* < 0.0001) and to the impact factor of the publishing journal (*r* = 0.7310; *p* < 0.0001). Of note, the items 10 (randomization: implementation) and 21 (generalizability) showed particularly high rates of non-fulfillment (87 and 85%). The majority of studies (95%) complied with item 2b (specific objectives or hypotheses), but strikingly objectives were mostly described vaguely. Other essential criteria such as sample size determination, definition of outcome parameters, and participant flow were only fulfilled weakly (51, 54, and 39%).

**Conclusions:** Over 20 years after its inception there is still weak adherence to the CONSORT statement. As a consequence, conclusions are hampered by inadequate planning and/or reporting. Particularly with respect to pathologies as ICH lacking clear, evidence-based guidelines adherence to the CONSORT statement might improve research quality in order to define valuable treatment strategies.

## Introduction

Intracranial hemorrhage (ICH) is the second most common cause of stroke; the overall global burden of hemorrhagic stroke (ICH and subarachnoid hemorrhage) including deaths and DALYs (disability-adjusted life years) is higher than in ischemic stroke, although ischemic stroke accounts for nearly twice as much number of incidents (1, 2). Of note, the DALYs lost due to hemorrhagic stroke are almost twice as high than those lost due to ischemic stroke (62,842,896 vs. 39,389,408 years, data for 2010) (1). Thus, ICH is a condition with huge impact on the patient's individual fate, but also accounts for enormous social and socio-economic consequences. There is still debate concerning the optimal medical therapy. Data on surgical treatment are conflicting: There is no consensus on the question which subgroup of patients actually benefits from a surgical intervention, if there is a benefit. Even the large STICH trials (3, 4) were not able to clarify this subgroup sufficiently and the conclusions especially drawn from the STICH trials are still debated controversially (5–7).

In general, problems arise from the fact that the term “ICH” summarizes heterogeneous entities that vary concerning genetic and lifestyle risk factors (8–10) and consecutively show different bleeding locations. Further timing of surgical intervention, indications for surgery, basic therapeutic strategies (e.g., blood pressure control) differ between the treating medical facilities. Some of these aspects may be hardly avoided planning a trial on ICH treatment.

There are also methodological aspects which have to be taken into account. Despite some disadvantages randomized controlled trials (RCT) still are regarded as the “gold standard” for a clinical research (11, 12). Treatment guidelines are usually based upon the results of RCTs or on systematic reviews/metaanalyses which in turn rest on the results acquired by RCTs and results of methodologically weak RCTs may alter their conclusions erroneously (13). Therefore, methodological quality is essential; this includes high quality of reporting (14). In 1998 the CONSORT (Consolidated Standards of Reporting Trials) statement (revised in 2001 and 2010) was published in order to provide a guideline for the reporting of RCTs (15–17). Basically, the CONSORT statement was intended to maximize research transparency in order to enable authors, reviewers, editors and finally readers to assess the methodology and consecutively to interpret the results properly. In fact, adherence to the CONSORT statement's criteria is associated with improvements in the quality of reports of RCTs (18–21). Concerning for example neurosurgical trials (which some of the ICH intervention trials are) both the quality of design and reporting is low (22).

## Objective

Here, we evaluated RCTs on primary ICH according to their fulfillment of the current CONSORT statement's criteria. Furthermore, we correlated the impact factor of the journal and the citation frequency with the ratio of CONSORT statement's criteria fulfillment. Thus, we aimed to analyze the transparency of reporting in ICH trials to identify targets in order to improve planning and reporting of ICH trials.

## Methods

### Study Design

We performed a cross sectional retrospective study (conducted at the University Hospital, RWTH Aachen University, Germany) and reported the data according to the STROBE statement (23). At first, we analyzed the literature on ICH from January 2014 to March 2017 (literature search: 04-23-2017) and included the timespan from January 2013 to December 2013 later on (literature search: 06-16-2017) to increase the number of articles available for inclusion. The abstracts were acquired using a Pubmed and a Cochrane database (including clinicaltrials.gov) search with the following search terms: [(cerebral hemorrhage) OR (hemorrhagic stroke) OR (intracerebral hemorrhage) OR ich OR (intracranial hemorrhage) OR (intracranial bleeding) OR (intracerebral hematoma) OR (intracranial hematoma)] AND [(randomized controlled) OR randomized OR RCT]. Two authors (KJ and AH) screened all abstracts for inclusion (applying the following inclusion criteria: randomized controlled clinical trial on patients with acute primary ICH). Therefore, abstracts of trials on secondary ICH (e.g., trauma-associated), experimental studies, review articles etc. were excluded (for details see Flowchart; Table 1). Discrepancies regarding the study allocation were discussed with a second author (AH). Afterwards, the eligible full text manuscripts were assessed for the implementation of the CONSORT criteria (see below).

**Table 1:** Flowchart showing the screening of abstracts and selection process for articles included and excluded in the current study.

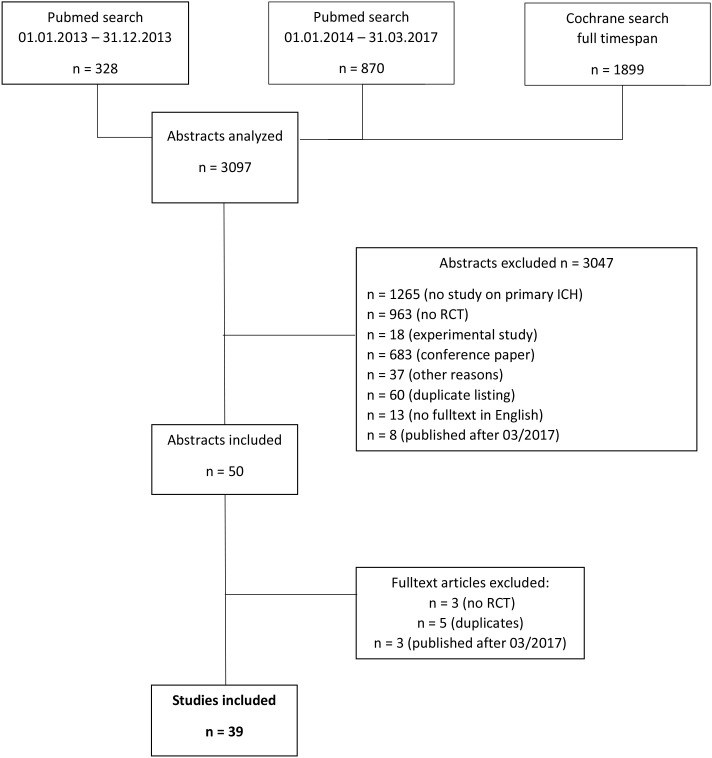

### Data Extraction

At first, a data sheet consisting of standardized categories representing the CONSORT items was elaborated (KJ, AK, and AH) to ensure an evaluation as objective as possible. The eligible full text articles (including available supplements) were examined for their fulfillment of the 37 CONSORT items by one author (KJ). About 10% of the items were cross-checked randomly by another author (AH). Further, in case of ambiguity the specific item was discussed between at least two of the authors (KJ, AH) and a decision was made together. Every item was classified as “fulfilled” (f), “not fulfilled” (nf) or “not applicable” (na). The classification “na” was feasible for items which are not mandatory, e.g., item 7b “When applicable, explanation of any interim analyses and stopping guidelines.”

Additionally, the risk of bias of each study was assessed using the revised Cochrane risk of bias (RoB) tool 2 (24, 25). For statistical purposes a “low risk” of bias was defined as a score of one, “some concerns” were defined as a score of two and a “high risk” was defined as a score of three. The sum scores (summation of the single domain scores) were documented and related with CONSORT criteria adherence.

The citation frequency and the impact factor of the journal (for the year the manuscript was published) were assessed using the Web of Science (Clarivate Analytics). Citation frequency per year was calculated to eliminate the bias derived from articles which are available for a longer period of time. Four studies (26–29) had to be excluded for additional analyses as it was not listed in the WEB of Science.

## Statistical Methods

We reported the percentages of CONSORT criteria adherence for each item. Additionally, summary statistics were calculated and graphically presented. Further, RoB scores were related with CONSORT criteria fulfillment. Citation frequency and citation frequency per year were correlated with impact factor and adherence of CONSORT criteria fulfillment: Due to non-linear relationships we computed the Spearman‘s rank-order correlation (computing the coefficient *r*) and reported the corresponding *p*-value. For better visualization due to non-linear relationship logarithmic axes were used. Therefore, for the graphical representation citation frequencies with the value zero were set to 0.001. All our statistical analyses were performed using GraphPad Prism 8.2.0 (GraphPad Prism Software Inc., La Jolla, CA, USA). A *p*-value of 0.05 was regarded statistically significant.

## Results

We analyzed a total of 3,097 abstracts; 1,157 had to be excluded due to the following reasons: no study on primary ICH (*n* = 1,265), no RCT (*n* = 963), experimental study (*n* = 18), duplicate listing (*n* = 60), no full text in English (*n* = 13), published after 03/2017 (*n* = 8), other reasons (*n* = 37). Because of duplicate listing five more studies had to be excluded. Thus, a total of 34 studies was available for full-text analysis. After full-text analysis three studies turned out not to be RCTs and three further studies were identified as published after March 2017. Therefore, 39 studies finally were analyzed (see Flowchart; Table 1) (4, 26–63). For further information on studies, specific interventions and outcome measures please see Supplemental Data File.

**Figure 1:**
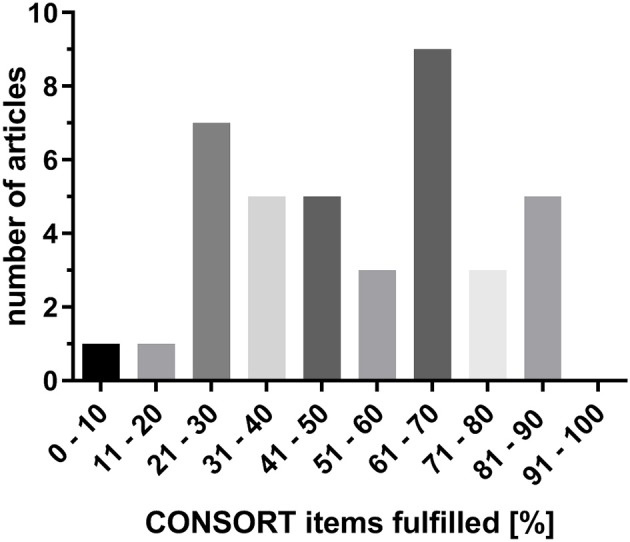
Percentages of CONSORT criteria adherence (considering all 37 items) for all studies included.

On average only 51% (±28%; *n* = 741) of the requested CONSORT items were fulfilled; 49% of the included studies (*n* = 19) complied with <50% of the CONSORT criteria (Figure 1). For each specific CONSORT item the percentage adherence of all 39 RCTs analyzed is shown in Tables 1, 2. Excluding the optional items and those only applicable for a portion of the studies (items 3b, 6b, 7b, 11b, 12b, 17b, 23, and 24) resulted in an adherence of 59% (±24%; *n* = 670).

**Table 2:** Table 1 shows the numbers and percentage adherence of the 39 RCTs analyzed to each CONSORT item.

		**Item description**	**Items fulfilled; percentage**
**Title and abstract**			
	1a	Identification as a randomized trial in the title	*n* = 16; 41.03%
	1b	Structured summary of trial design, methods, results, and conclusions	*n* = 15; 38.46%
**Introduction**			
Background and objectives	2a	Scientific background and explanation of rationale	*n* = 37; 94.87%
	2b	Specific objectives or hypotheses	*n* = 37; 94.87%
**Methods**			
Trial design	3a	Description of trial design (such as parallel, factorial) including allocation ratio	*n* = 31; 79.49%
	3b	Important changes to methods after trial commencement (such as eligibility criteria), with reasons	*n* = 2; 5%
Participants	4a	Eligibility criteria for participants	*n* = 34; 87.18%
	4b	Settings and locations where the data were collected	*n* = 28; 71.79%
Interventions	5	The interventions for each group with sufficient details to allow replication, including how and when they were actually administered	*n* = 35; 82.05%
Outcomes	6a	Completely defined pre-specified primary and secondary outcome measures, including how and when they were assessed	*n* = 21; 53.85%
	6b	Any changes to trial outcomes after the trial commenced, with reasons	*n* = 1; 2.56%
Sample size	7a	How sample size was determined	*n* = 20; 51.28%
	7b	When applicable, explanation of any interim analyses and stopping guidelines	*n* = 4; 10.26%
**Randomization**			
Sequence generation	8a	Method used to generate the random allocation sequence	*n* = 28; 71.79%
	8b	Type of randomization; details of any restriction (such as blocking and block size)	n = 8; 20.51%
Allocation concealment mechanism	9	Mechanism used to implement the random allocation sequence (such as sequentially numbered containers), describing any steps taken to conceal the sequence until interventions were assigned	*n* = 11; 28.21%
Implementation	10	Who generated the random allocation sequence, who enrolled participants, and who assigned participants to interventions	*n* = 5; 12.82%
Blinding	11a	If done, who was blinded after assignment to interventions (for example, participants, care providers, those assessing outcomes) and how	*n* = 24; 61.54%
	11b	If relevant, description of the similarity of interventions	*n* = 2; 5.13%
Statistical methods	12a	Statistical methods used to compare groups for primary and secondary outcomes	*n* = 23; 58.97%
	12b	Methods for additional analyses, such as subgroup analyses and adjusted analyses	*n* = 16; 41.03%
**Results**			
Participant flow (a diagram is strongly recommended)	13a	For each group, the numbers of participants who were randomly assigned, received intended treatment, and were analyzed for the primary outcome	*n* = 15; 38.46%
	13b	For each group, losses and exclusions after randomization, together with reasons	*n* = 16; 41.03%
Recruitment	14a	Dates defining the periods of recruitment and follow-up	*n* = 33; 84.62%
	14b	Why the trial ended or was stopped	*n* = 18; 46.15%
Baseline data	15	A table showing baseline demographic and clinical characteristics for each group	*n* = 32; 82.05%
	16	For each group, number of participants (denominator) included in each analysis and whether the analysis was by original assigned groups	*n* = 23; 58.97%
Outcomes and estimation	17a	For each primary and secondary outcome, results for each group, and the estimated effect size and its precision (such as 95% confidence interval)	*n* = 27; 69.23%
	17b	For binary outcomes, presentation of both absolute and relative effect sizes is recommended	*n* = 20; 51.28%
Ancillary analyses	18	Results of any other analyses performed, including subgroup analyses and adjusted analyses, distinguishing pre-specified from exploratory	*n* = 20; 51.28%
Harms	19	All important harms or unintended effects in each group (for specific guidance see CONSORT for harms)	*n* = 20; 51.28%
**Discussion**			
Limitations	20	Trial limitations, addressing sources of potential bias, imprecision, and, if relevant, multiplicity of analyses	*n* = 31; 79.49%
Generalizability	21	Generalizability (external validity, applicability) of the trial findings	*n* = 6; 15.38%
Interpretation	22	Interpretation consistent with results, balancing benefits and harms, and considering other relevant evidence	*n* = 39; 100%
**Other information**			
Registration	23	Registration number and name of trial registry	*n* = 15; 38.46%
Protocol	24	Where the full trial protocol can be accessed, if available	*n* = 11; 28.21%
Funding	25	Sources of funding and other support (such as supply of drugs), role of funders	*n* = 20; 51.28%
		A total of	*n* = 741; 51.35%

Of note, the items 10 (randomization: implementation) and 21 (generalizability) showed particularly high rates of non-fulfillment (87 and 85%). The majority of studies (95%) complied with item 2b (specific objectives or hypotheses), but strikingly objectives were mostly described vaguely. Applying a strict interpretation of item 2b (demanding a clear hypothesis including outcome specification) only 23% would have fulfilled this item. Other essential criteria such as sample size determination, definition of outcome parameters and participant flow were only fulfilled weakly (51, 54, and 39%). Less than a 50% adherence was demonstrated for 43% (*n* = 16) of the CONSORT items (excluding the optional ones −3b, 6b, 7b, 11b, 12b, 17b, 23, and 24-: in 31% of the mandatory items –*n* = 9- a fulfillment rate of <50% was seen) (Table 2).

**Figure 2:**
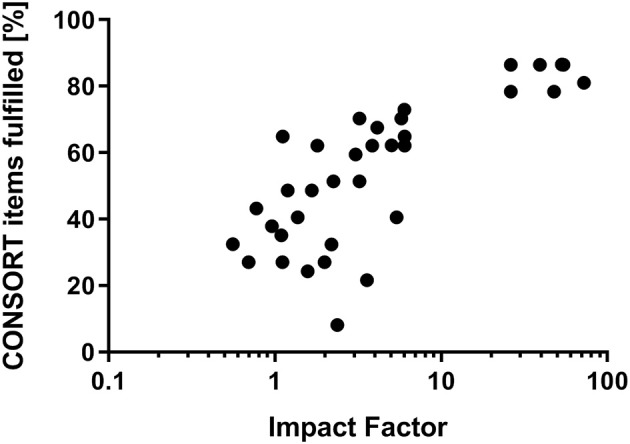
Correlation of journals' impact factor and fulfillment of CONSORT checklist. Due to non-linear relationship a logarithmic x-axis was chosen.

**Figure 3:**
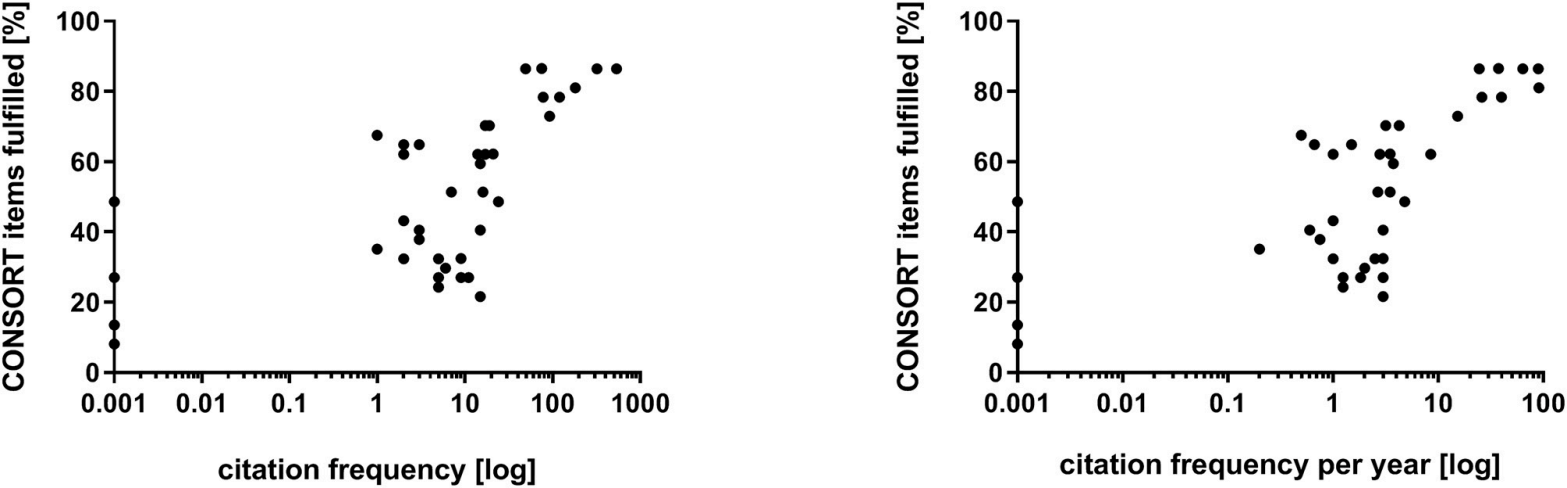
Correlation of citation frequency **(Left)**, respectively, citation frequency per year **(Right)** and fulfillment of CONSORT checklist. Due to non-linear relationship a logarithmic x-axis was chosen. Therefore, citation frequencies with the value zero were set to 0.001.

**Figure 4:**
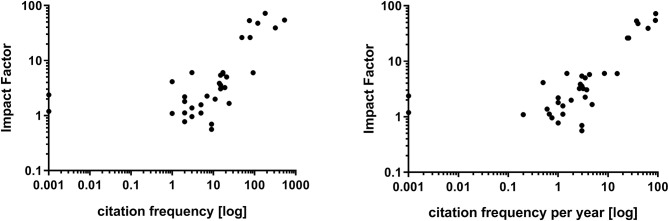
Correlation of citation frequency **(Left)**, respectively, citation frequency per year **(Right)** and journals' impact factor. For better visualization logarithmic axes were chosen. Therefore, citation frequencies with the value zero were set to 0.001.

Examining the relationship of the journals' impact factor with the fulfillment of CONSORT criteria a distinct correlation (*r* = 0.7664; *p* < 0.0001) was shown (Figure 2). Citation frequency was slightly related to ratio of CONSORT item fulfillment (*r* = 0.6605; *p* = 0.0001; expressed as citation frequency per year: *r* = 0.6747; *p* < 0.0001) (Figure 3). The citation frequency was highly associated with the impact factor of the publishing journal (*r* = 0.7297; *p* < 0.0001; expressed as citation frequency per year: *r* = 0.7310; *p* < 0.0001) (Figure 4).

Indeed, the CONSORT statements are guidance for authors, editors, and reviewers to improve transparency, and although each item should theoretically be reported, each item cannot be considered as having the same importance. For example, items related to “randomization,” which are essential to evaluate the risk of bias of the study, could be considered more important than items related to “scientific background and explanation of rationale.” It is far more relevant to report the completeness of reporting for each item separately and explore its evolution over time.

**Figure 5:**
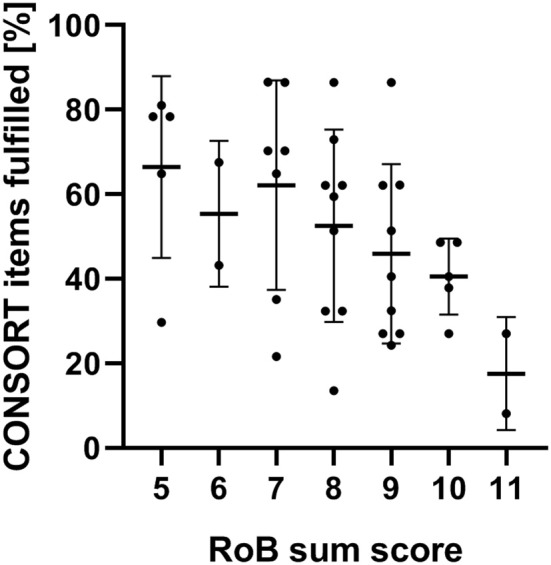
Risk of bias sum scores (assessed via RoB 2) and the corresponding CONSORT criteria adherence for all studies included.

The risk of bias tool (25) and its revised version (24) aim to judge the risk of bias in randomized trials according to five domains (Domain I: Randomization Progress; Domain II: Deviations from intended interventions; Domain III: Missing outcome data; Domain IV: Measurement of the outcome; Domain V: Selection of the reported results). We analyzed the included studies according to RoB 2: The RoB sum score and CONSORT criteria fulfillment correlated very weakly (*r* = 0.4758; *p* = 0.0022). A graphic illustration of the RoB sum scores is shown in Figure 5.

## Discussion

Despite the clinical urge for evidence based guidelines concerning the treatment of ICH there is still very weak adherence to the CONSORT statement (average: 51%). The impact factor of the publishing journal and the citation frequency correlate with the ratio of CONSORT item fulfillment. There are some important items, which in general demonstrate low adherence rates (e.g., details of randomization). Of note, substantial items (such as sample size determination, definition of outcome parameters and participant flow) were only fulfilled weakly (55, 61, and 45%).

In 2011 Kiehna et al. reported a low CONSORT criteria adherence score of neurosurgical RCTs (published 2006–2007 in: Journal of Neurosurgery; Neurosurgery; Surgical Neurology; Journal of Neurology, Neurosurgery and Psychiatry; Acta Neurochirurgica) compared to the three leading general medicine journals (Journal of the American Medical Association; Lancet; The New England Journal of Medicine) (mean score of 26.4 vs. 41 out of 44) (64). Similarly, Mansouri et al. published an analysis on RCTs in neurosurgery (2000–2014) using specific search terms (22). It was shown that the reporting quality in general was low (median CONSORT score of 36 out of 44) and that specific items such as blinding, sample size calculation, allocation concealment and protocol implementation were reported rarely. Sample size calculation, which is essential for an RCT to produce a relevant conclusion, was documented in only 20–34.2% (depending on subspecialty). The reporting of this specific actually has improved (reporting in 51% of the studies included). But generally, in line with the prior results –more than 20 years after the first publication of the CONSORT statement- we demonstrated substantial paucities regarding the planning and reporting of RCTs on ICH. In fact, there are some studies with a reporting at a high quality level, but lack fundamental issues such as a randomization, sample size calculation, and transparent participant flow allowing the reader to understand the number of patients analyzed including drop-outs and specific reasons. Thus, not only the mere ratio of adherence has to be taken into account. This is supported by a statement of the member of the CONSORT steering group:”…although each item should theoretically be reported, each item cannot be considered as having the same importance” (65).

However, in contrast to the above mentioned studies our analysis was based on a specific pathology, ICH, and was not only limited to RCTs published in neurosurgical journals but also incorporates RCTs published in journals focused on other areas such as critical care or general medicine.

ICH is the second most common cause of stroke and accounts for a high mortality and morbidity Still the median case fatality at 1 month is as high 40.4% (2). However, due to the heterogeneous disease patterns (in the framework of different localizations, risk factors, clinical presentations etc.) it is challenging to conceive an RCT representing the entire spectrum of ICH. Therefore, analyses of subgroups (which has already been done) may be feasible to increase the evidence for ICH treatment. Additionally, a special CONSORT extension addressing non-pharmacological interventions was published in 2017 (66), which has not been taken into account as it was published in July 2017 (studies have been included from 01/2013 to 03/2017). However, many items –especially the essential ones such as randomization, sample size calculation, definition of outcome measures-overlap.

For other major specialties such as anesthesiology, critical care, or surgery the CONSORT criteria adherence has also been analyzed (67–70). In general, all studies report a demand for improvement of planning and reporting. Even if the analyses are limited to high-ranked journals non-adherence to the CONSORT statement (especially concerning methodological quality domains) is frequent (71, 72); albeit these studies only examined the abstracts published. Journal endorsement of the CONSORT statement was attested to improve the quality of reporting in terms of completeness and transparency (20, 21). However, there are still scarcities and inaccuracies as adherence is not always monitored rigorously (21).

Specific items –regardless of the CONSORT criteria- have been evaluated extensively, such as intervention description and outcome measures (73, 74): It has been shown, that only 13% of articles published in the top five medical journals (New England Journal of Medicine, Lancet, JAMA, The BMJ, and Annals of Internal Medicine) October 2015 to January 2016 were “perfectly” reported, meaning that the original primary and secondary outcome measures were reported as initially prespecified (74). The authors have found that 354 outcome were not reported and that 357 outcomes were reported that in the forefront had not laid down in the protocol (74). Concerning intervention of 137 interventions reported in 2009 in the leading 6 medical journals only 53 (39%) were adequately described (73). Again, this may influence the interpretation of the results presented misleadingly, replication of trials is hampered and interventions intended as therapeutic procedure may not be conducted properly. In conclusion, inaccuracies in planning and reporting of RCTs are frequent and can bias their interpretation severely. The endorsement of the CONSORT statement may improve the reporting of RCTs.

There are enormous problems at different stages of research, which are not always solved by adherence to the CONSORT criteria. For example, when planning a new trial only 11 out of 24 authors were not aware of the Cochrane reviews that already existed (75). Methodological flaws, such as inadequate or unclear concealment of treatment allocation (18, respectively, 26% evaluating 234 trials published in the major general medical) (76), are frequent. These are only some aspects which result in the “production and reporting of avoidable waste in research” (77). Due to limited resources, ethical issues and consequences concerning clinical algorithms the problem of inaccurately planned and reported research must be tackled. Adherence to the CONSORT guidelines may not be the entire solution but at least a helpful start. Here, we present the data on 39 RCTs concerning ICH. Our analysis is limited to the fact that the interpretation of the CONSORT criteria sometimes is objective and the judgement between different observers may vary. We performed a cross-check of 10% of the data and uncertainties were discussed between at least two of the authors. Anyway, there is bias derived from the fact, that mainly one author was responsible for the judgement of adherence to the CONSORT criteria. Further, some of the criteria are not binary parameters but leave some margin for interpretation. Again, judgement may depend distinctly on the individual observer. Further, our analysis does not weight the specific items.

## Conclusion

Over 20 years after its inception there is still weak adherence to the CONSORT statement: Inadequate planning and/or reporting is frequent and essential information such as sample size calculation and clear definitions of outcome measures are often missing. As a consequence, conclusions based on these trials are regularly hampered. Reviewers and readers are not able to classify the results properly. Due to incomplete reporting scientist are not able to replicate the trials. Particularly with respect to pathologies as ICH lacking clear, evidence-based guidelines endorsement of the CONSORT statement and a consequent audit of its adherence might improve research quality in order to define valuable treatment strategies.

## Data Availability

The data analyzed during this study are included in this published article (references of analyzed articles, ratios of adherence). Additional information (data on citation frequencies, impact factors, ratios of non-fulfillment…) is available from the corresponding author on reasonable request.

## Author's Note

Portions of this work were presented in abstract form at the “Arbeitstagung NeuroIntensiv Medizin—ANIM” 2019 in Berlin, Germany, January 18, 2019.

## Author Contributions

AH conceived and designed the study. AK and MC helped to elaborate a revised study plan. KJ, AK, and AH elaborated a data sheet of standardized categories representing the CONSORT items. KJ and AH screened the abstracts and analyzed the data. KJ examined the full-text articles and drafted the first version of the manuscript. AH cross-checked 10% of the screened items. In case of ambiguity the specific item was discussed between KJ and AH. AH, AK, MC, and HC critically revised interpretation of the data and revised the first draft of the manuscript.

### Conflict of Interest Statement

The authors declare that the research was conducted in the absence of any commercial or financial relationships that could be construed as a potential conflict of interest.

## Supplementary Material

Supplemental Data File Summary of the studies included: Title, Journal (including year of publication), intervention and primary outcome.
