# Supplementary material for: Randomized Controlled Trials on Intracerebral Hemorrhage: A Cross Sectional Retrospective Analysis of CONSORT Item Adherence
Source: Front Neurol. 2019 Sep 20;10:991. doi: 10.3389/fneur.2019.00991 (PMC6763943; doi:10.3389/fneur.2019.00991)
Supplement: Supplemental Data File — Summary of the studies included: Title, Journal (including year of publication), intervention and primary outcome. [file Table_1.DOCX]

| **Name of study** | **Journal - Year of publication** | **Interventions** | **Primary outcome** |
| --- | --- | --- | --- |
| Endoscope-Assisted Keyhole Technique for Hypertensive Cerebral Hemorrhage in Elderly Patients: A Randomized Controlled Study in 184 Patients | Journal of Turkish Neurosurgery - 2016 | TG (n=93): operation with an endoscope-assisted keyhole technique.  CG (n=91): traditional hematoma drainage. | Six-month effect on the activities of daily living (ADL), ADL-score. |
| Microsurgical treatment assisted by intraoperative ultrasound localization: A controlled trial in patients with hypertensive basal ganglia hemorrhage | British Journal of Neurosurgery - 2014 | TG (n=51): Microsurgical treatment assisted with intraoperative ultrasound localization.  CG (n=56): Conventional microsurgery. | Not particularly defined.  Extention of hematoma evacuation and brain edema. |
| Benefits of a frame-based stereotactic surgical planning system for the treatment of spontaneous intracerebral haematomas | Journal of International Medical Research - 2013 | TG (n=30): ICH removal with a frame-based stereotactic surgical planning system  CG (n=35): ICH removal without a frame-based stereotactic surgical planning system | Not particularly defined.  Duration of hematoma evacuation and number of urokinase injections were used as outcome parameter. |
| Effect of local mild hypothermia on regional cerebral blood flow in patients with acute intracerebral hemorrhage assessed by 99mTc-ECD SPECT imaging | Journal of X-Ray Science and Technology - 2015 | TG (n=19): local mild hypothermia with conventional mannitol  CG (n=17): standard therapy with conventional mannitol | Not particularly defined.  National Institutes of Health Stroke Scale (NIHSS) at 14 and 21 days after therapy.  Glasgow Coma Scale (GCS).  Brain SPECT 7 days post-therapy. |
| Activating Blood Circulation to Remove Stasis Treatment of Hypertensive Intracerebral Hemorrhage: A Multi-Center Prospective Randomized Open-Label Blinded-Endpoint Trial | Chinese Journal of Integrative Medicine - 2016 | TG (n=102): Conventional treatment plus Chinese herbal therapeutic regimen of activating blood circulation.  CG (n=108): Conventional treatment only. | Degree of disability 3 months after entering the trial (measured by modified Rankin Scale). |
| Angiotensin II receptor blockers following intravenous nicardipine administration to lower blood pressure in patients with hypertensive intracerebral hemorrhage: a prospective randomized study | Blood pressure monitoring - 2017 | TG (n=15): 20mg azilsartan  CG (n=15): 8mg candesartan  Both groups recieved intravenous nicardipine for 24-48h before start of azilsartan/candesartan. | Frequency of hematoma expansion. |
| Decreased risk of secondary brain herniation with intracranial pressure monitoring in patients with hamorrhagic stroke | BMC Anesthesiology - 2014 | TG (n=46): ICP monitoring during general anesthesia  CG (n=44): standard/general anesthesia without ICP monitoring. | Hematoma enlargement and secondary brain herniation 1 month after study onset. |
| Blood pressure control in ultra-early basal ganglia intracerebral hemorrhage | European Review for Medial and Pharmacological Sciences - 2015 | TG (n=60): Systolic blood pressure control for 24h among 130-140 mmHg.  CG (n=60): Systolic blood pressure control for 24h among 160-180 mmHg. | Not particularly defined.  National Institutes of Health neurological deficit score (NIHSS) after 24 hours and 14 days of treatment.  Hematoma volume 24 hours, 5 and 14 days after admission.  Serum MMP-9 level. |
| Tranexamic Acid for Spontaneous Intracerebral Hemorrhage: A Randomized Controlled Pilot Trial | Journal of Stroke and Cerebrovascular Diseases - 2014 | TG (n=16): standard care plus 1g bolus of tranexamic acid intravenous and 1g of tranexamic acid as a infusion for a period of 8 hours.  CG (n=8): standard care plus a matching placebo. | Feasibility of recruiting to a definitive trial/study (trial feasibility). |
| Efficacy and safety of penetration acupuncture on head for acute intracerebral hemorrhage | Medicine - 2016 | 82 patients, TG (n=42): acupuncture treatment on head (24 sessions over 4 weeks) plus conventional treatment, CG (n=40): conventional treatment only. 3 Month follow up. | Clinical Neurological Function Deficit Scale (CNFDS) at day 7, 14, 28, 60, 90 after enrollment. Barthel Index (BI) at day 28, 60, 90 after enrollment. |
| Minimally Invasive Procedures Reduced the Damages to Motor Function in Patients with Thalamic Hematoma: Observed by Motor Evoked Potential and Diffusion Tensor Imaging | Journal of Stroke and Cerebrovascular Diseases - 2013 | TG (n=20): Minimally invasive group.  CG (n=23): Medical treatment group.  Both groups also recieved standard medical therapy | Not particularly defined.  Diffusion tensor imaging 2 weeks after surgical procedures.  Integrity of corticospinal tract.  Motor-evoked potential on admission and 2 weeks after.  Modified National Institute of Health Stroke Scale Score. |
| Decompressive craniectomy and expansive duraplasty with evacuation of hypertensive intracerebral hematoma, a randomized controlled trial. | Neurosurgical Review - 2017 | TG (n=20): Hematoma evacuation together with decompressive craniectomy and expansive duraplasty.  CG (n=20): Standard hematoma evacuation | Not particularly defined.  Modified Rankin Scale and Glasgow Outcome Scale at 6 months‘ follow up. |
| The Clinical Study of Stereotactic Microsurgery | Cell Biochemistry and Biophysics - 2013 | TG (n=50): Treatment with stereotactic microsurgery (Leksell-G system).  CG (n=50): Treatment with standard microsurgery. | Not particularly defined.  Disease classification.  Hematoma volume.  Efficacy of treatment method. |
| The Clinical Effect of Deferoxamine Mesylate on Edema after Intracerebral Hemorrhage - 2015 | PLOS ONE - 2015 | TG (n=21): standard treatment with 32 mg/kg deferoxamine mesylate intravenous (daily) for 3 days  CG (n=21): standard treatment without deferoxamine mesylate | Relative endema volume on the fifteenth day (or discharge). |
| Effect of acetylsalicylic acid usage and platelet transfusion on postoperative hemorrhage and activities of daily living in patients with acute intracerebral hemorrhage | Journal of Neurosurgery - 2013 | TG: Patients with acetylsalicylic acid therapy already before hospitalization. If sensitive to therapy: randomly assigned to either no frozen apheresis platelets transfusion or 1 dose before surgery or 2 doses (1 before and 1 after surgery).  CG: Patients without acetylsalicylic acid therapy before hospitalization  Both groups: emergency craniotomy | Postoperative hemorrhage rate and average postoperative hemorrhage volume. |
| A randomized controlled study comparing omeprazole and cimetidine for the prophylaxis of stress-related upper gastrointestinal bleeding in patients with intracerebral hemorrhage | Journal of Neurosurgery - 2013 | TG1 (n=58): 40mg omeprazole intravenous every 12 hours  TG2 (n=54): 300mg cimetidine intravenous every 6 hours  CG (n=53): placebo | Occurence of upper gastrointestinal bleeding within 15 days of ictus or death within 30 days of ictus. |
| Acute Intracranial Hemorrhage in CT: Benefits of Sinogram-Affirmed Iterative Reconstruction Techniques | American Journal of Neuroradiology - 2014 | TG (n=40): CT at 260 mAs  (new scan parameter)  CG (n=54): CT at 340 mAS (standard scan parameter)  Looking for image quality, detectability of ICH and reduction of radiation dose in brain CT scanning | Not particularly defined.  Image quality and identifiable properties of cerebral hemorrhage. |
| Effects of celecoxib on hematoma and edema volumes in primary intracerebral hemorrhage: a multicenter randomized controlled trial | European Journal of Neurology - 2013 | TG (n=20): 400mg celecoxib twice a day for 14 days  CG (n=24): standard medical treatment | Change in the volume of perihematoma endema from the 1st to the 7th day. |
| PG2 for patients with acute spontaneous intracerebral hemorrhage: a double-blind, randomized, placebo-controlled study | Scientific reports - 2017 | Total of 61 patients. (TG 30 patients; CG 31 patients). TG recieved 3 doses of PG2 (500mg, iv) per week for two weeks. Control group received placebo. | Change of Glasgow outcome scale and/or Modified Rankin scale scores after PG2 treatment/placebo.  (baseline, 7, 28, 84 days after PG2/placebo administation) |
| Blood pressure reduction does not reduce perihematoma oxygenation: a CT perfusion study | Journal of Cerebral Blood Flow and Metabolism - 2014 | TG (n=34): systolic blood pressure <150 mmHg  CG (n=31): systolic blood pressure <180 mmHg  Both groups: CT perfusion imaging 2 hours after randomization 🡪 perihematoma oxygenation. | Not particularly defined.  Perihematoma oxygenation.  Clinical/neurological deficits with National Institutes of Health Stroke Scale.  Functional disability with modified Rankin Scale. |
| Randomized Controlled Trial of Early Rehabilitation After Intracerebral Hemorrhage Stroke | Stroke - 2014 | TG (n=122): standard care plus early rehabilitation  CG (n=121): standard care | Survival 6 months after stroke |
| The Intracerebral Hemorrhage Acutely Decreasing Arterial Pressure Trial | Stroke - 2013 | TG (n=39): Systolic blood pressure <150mmHg  CG (n=36): Systolic blood pressure < 180 mmHg  Both groups: computed tomography perfusion imaging 2 hours postrandomization. | Perihematoma relative blood flow. |
| Perioperative Antihypertensive Treatment in Patients With Spontaneous Intracerebral Hemorrhage | Stroke - 2016 | 201 patients, TG (n=100): perioperative intensive antihypertensive treatment (syst. blood pressure between 120 and 140 mmHg). CG (n=101): syst. blood pressure between 140 and 180 mmHg for 7 days. | Rate of rehemorrhage 7 days after surgery. |
| ICES (Intraoperative Stereotactic Computed Tomography-Guided Endoscopic Surgery) for Brain Hemorrhage | Stroke - 2016 | Endoscopic surgery (TG n=18) versus standard madical management (CG n=6+36). | Primary safety outcomes: 30 day rate of mortality, the 7 day rate of procedure-related mortality, 30 day rate of bacterial brain infection, rate of symptomatic bleeding within 72 hours.  Primary efficacy outcome: 180 day dichotomized mRS score 0 to 3 versus 4 to 6. |
| Safety and efficacy of minimally invasive surgery plus alteplase in intracerebral haemorrhage evacuation (MISTIE): a randomised, controlled, open-label, phase 2 trial | The Lancet Neurology - 2016 | Standard medical care versus image-guided minimally invasive surgery plus alteplase to remove clots using surgical aspiration followed by alteplase clot irrigation. | 30 day mortality, 7 day procedure-related mortality, 72h symptomatic bleeding, 30 day brain infections |
| Fresh frozen plasma versus prothrombin complex concentrate in patients with intracranial haemorrhage related to vitamin K antagonists (INCH): a randomised trial | The Lancet Neurology - 2016 | Patients with VKA-ICH, INR at least 2.0  TG: 20ml/kg intravenous FFP. CG: 30 IU/kg of intravenous four-factor PCC. Both groups within 1h after initial cerebral CT scan. | Effect of the investigational product on anticoagulation reversal, measured as the proportion of patients with an INR of 1.2 or lower at 3h after the beginning of treatment. |
| Early surgery versus initial conservative treatment in patients with spontaneous supratentorial lobar intracerebral haematomas (STICH II): a randomised trial | The Lancet - 2013 | TG (n=305): Early surgical haematoma evacuation within 12 hours of randomisation plus medical treatment  CG (n=292): initial medical treatment | Prognosis-based dichotomised outcome of the 8 point Extended Glasgow Coma Scale at 6 months. |
| Platelet transfusion versus standard care after acute stroke due to sponataneous cerebral haemorrhage associated with antiplatelet therapy (PATCH): a randomised, open-label, phase 3 trial | The Lancet - 2016 | TG (n=97): standard care with platelet transfusion within 90min of diagnostic.  CG (n=93): standard care | Difference in functional outcome scored with the modified Rankin scale at 3 months after randomisation. |
| Rapid Blood-Pressure Lowering in Patients with Acute Intracerebral Hemorrhage | The New England Journal of Medicine - 2013 | TG (n=1399): systolic blood pressure <140 mmHg for 7 days.  CG (n=1430): systolic blood pressure < 180 mmHg for 7 days. | Death or major disability at 90 days. |
| Intensive Blood-Pressure Lowering in Patients with Acute Cerebral Hemorrhage | The New England Journal of Medicine - 2016 | TG (n=500): intensive blood pressure lowering, systolic blood pressure: 110 to139 mmHg  CG (n=500): systolic blood pressure: 140 to 179 mmHg | Death or disability (modified Rankin scale score of 4 to 6) at 3 months after randomization. |
| Tranexamic Acid as Antifibrinolytic Agent in Non Traumatic Intracerebral Hemorrhages | The Malaysian Journal of Medical Sciences - 2015 | TG (n=15): Treatment with Tranexamic Acid as antifibrinolytic Agent. Intravenous bolus of 1g, followed by infusion 1g/hour for 8 hours.  CG (n=15): standard treatment, placebo | Effect of TXA on hematoma growth after 24 hours. |
| A French multicenter randomised trial comparing two dose-regimens of prothrombin complex concentrates in urgent anticoagulation reversal | Critical Care – 2013 | Patients with vitamin K antagonist associated intracranial haemorrhage  TG (n=30): 40 IU/kg of 4-factor PCC as quickly as possible intravenously (in emergency room).  CG (n=29): 25 IU/kg of 4-factor PCC as quickly as possible intravenously (in emergency room).  (PCC = Prothrombin complex concentrates) | International normalized ratio (INR) 10 minutes after the end of 4-factor PCC infusion. |
| MMP-9, brain endema, and length of hospital stay of patients with spontaneous supratentorial intracerebral hemorrhage after hematoma evacuation along with the administration of tigecycline | Medical Journal of Indonesia – 2016 | TG (n=35): 100mg of tigecycline intravenously as prophylactic antibiotics before surgery  CG (n=37): 2g of fosfomycine intravenously as prophylactic antibiotics before surgery | Not particularly defined.  MMP-9 plasma level on the day 1 and the day 7.  Brain endema on day 7.  Length of hospital stay.  (MMP-9 = matrix metalloproteinases-9) |
| Clinical effect of minimally invasive intracranial hematoma in treating hypertensive cerebral hemorrhage | Pakistan Journal of Medical Sciences – 2016 | TG (n=78): minimally invasive evacuation of hematoma  CG (n=78): conventional craniotomy evacuation of hematoma | Not particularly defined.  Neurological impairment score (5^th^, 10^th^, 15^th^ day).  Barthel Index  Operation time, hematoma disappearance time, number of patients with hematoma cleared on first attempt |
| Thrombolytic removal of intraventricular haemorrhage in treatment of severe stroke: results of the randomized, multicenter, multiregion, placebo-controlled CLEAR III trial | Lancet – 2017 | TG (n=249): up to 12 doses (8 hours apart) of 1mg alteplase via the extraventricular drain.  CG (n=251): up to 12 doses (8 hours apart) of 1mg saline via the extraventricular drain. | Good functional outcome, defined as a modified Rankin Scale score of 3 or less at 180 days. |
| Prevalence of Shunt Dependency and Clinical Outcome in Patients with Massive Intraventricular Haemorrhage Treated with Endoscopic Washout and External Ventricular Drainage | The Malaysian Journal of Medical Sciences – 2017 | TG (n=19): Surgical treatment in form of an endoscopic Washout plus placement of an external ventricular drain.  CG (n=20): Only surgical treatment: placement of an external ventricular drainage. | Clinical outcome (modified Rankin Scale score) at six months after the procedure. |
| Repetitive hyperbaric oxygen treatment increase insulin sensitivity in diabetes patients with acute intracerebral hemorrhage | Neuropsychiatric Disease and Treatment – 2017 | TG (n=23): Hyperbaric oxygen therapy  CG (n=29): Normobaric oxygen therapy | National Institutes of Health Stroke Scale ten days and one month after onset. |
| Neuronavigation-Assisted Aspiration and Electro-Acupuncture for Hypertensive Putaminal Hemorrhage: A Suitable Technique on Hemiplegia Rehabilitation | Journal of Turkish Neurosurgery - 2016 | Group 1 (n=60): Neuronavigation-assisted aspiration and Electro-acupuncture  Group 2 (n=60): Neuronavigation-assisted aspiration  Group 3 (n=60): Electro-acupuncture  Group 4 (n=60): Conservative therapy consisting solely of medications | Not particularly defined.  Motor recovery (Fugl-Meyer Assessment of Physical Performance, Modified Ashwort Scale, Barthel Index) on weeks zero and eight. |
| Observation on the application of emergency green channel in clinical rescue of acute cerebral haermorrhage patients | Biomedical Research – 2017 | TG (n=34): Patients in prehospital emergency green channel  CG (n=34): No prehospital emergency green channel | Not particularly defined.  Treatment time, clinical curative effect, Barthel Index, Fugl-Meyer Assessment score. |
